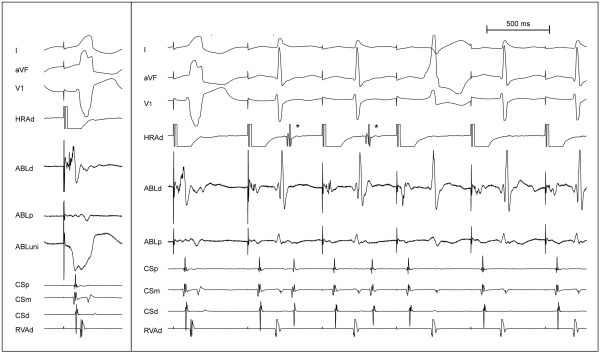# Sequential Anterograde and Retrograde Conduction Block during Radiofrequency Ablation of an Accessory Pathway

**DOI:** 10.1016/s0972-6292(16)30649-0

**Published:** 2013-08-01

**Authors:** Chandramohan Ramasamy, Rahul Ramteke, Jayaraman Balachander, Raja Selvaraj

**Affiliations:** Department of Cardiology, Jawaharlal Institute of Postgraduate Medical Education and Research

**Keywords:** Accessory Pathway, Radiofrequency Ablation, Anterograde and Retrograde Conduction Block

## Abstract

We present an interesting image showing sequential loss of anterograde, and subsequently, retrograde conduction during radiofrequency ablation of an accessory pathway. We discuss the possible mechanisms and prior literature concerning this interesting finding.

A young male with a history of recurrent palpitations and preexcitation in the electrocardiogram underwent an electrophysiology study. A right free wall accessory pathway with anterograde and retrograde conduction and inducible orthodromic atrioventricular reentrant tachycardia were demonstrated. Mapping was performed with a 4 mm tip ablation catheter (Therapy, St Jude Medical, Minnesota) during atrial pacing and showed early ventricular activation at 10'O clock on the tricuspid annulus. At the best site, local bipolar ventricular activation was about 50 ms ahead of surface delta wave onset, a sharp pathway potential was seen and unipolar electrogram showed a qS pattern (Figure 1, panel A).

Loss of preexcitation was seen two seconds after radiofrequency ablation was started (Figure 1, panel B). However, an atrial activation closely following the ventricular activation was seen for two beats after the loss of preexcitation (marked with asterisks). The pattern of atrial activation being earliest in the ablation catheter, appearance after loss of anterograde pathway conduction, similarity of the ventriculo-atrial (VA) interval to the VA interval during induced orthodromic reentrant tachycardia and disappearance after two beats pointed to these being atrial echo beats due to retrograde conduction through the pathway, and this disappeared in another two beats.

Differential effects on anterograde and retrograde accessory pathway conduction by radiofrequency ablation [1-3] and cryoablation [4] have been described before. Anatomical longitudinal dissociation of the accessory pathway with ablation of anterograde and retrograde conduction at different locations has been suggested as a mechanism [1,3]. However, sequential loss of anterograde conduction followed by retrograde conduction after two beats without catheter movement in our patient makes this unlikely. Impedance mismatch because of change in the mass of conducting tissue from the accessory pathway to the atrium or ventricle can account for conduction block. Alteration in this during ablation of an accessory pathway can explain unidirectional conduction block [4]. This could also explain the new appearance of preexcitation after radiofrequency ablation in patients with concealed conduction [5]. We think that this is the most likely explanation for the transient unidirectional block seen in our patient. While serving as a good illustration of this phenomenon because the loss of anterograde conduction is followed by evidence of preserved retrograde conduction in successive beats, we think this also serves as a reminder of the need to be aware of and test for the possibility of persistent retrograde conduction after loss of preexcitation during ablation of an accessory pathway.

## Figures and Tables

**Figure 1:**